# Takotsubo Cardiomyopathy: One More Angiographic Evidence of Microvascular Dysfunction

**DOI:** 10.1155/2018/5281485

**Published:** 2018-03-14

**Authors:** Marco Loffi, Andrea Santangelo, Martin Kozel, Viktor Kocka, Tomas Budesinsky, Libor Lisa, Petr Tousek

**Affiliations:** ^1^Cardiology Clinic, UO Malattie Cardiovascolari, Fondazione IRCCS, Ospedale Maggiore Policlinico, Dipartimento di Scienze mediche e di Comunità, University of Milan, 20122 Milan, Italy; ^2^Department of Cardiology, University of Palermo, 90127 Palermo, Italy; ^3^Cardiocenter, Third Faculty of Medicine, Charles University and University Hospital Kralovske Vinohrady, 10000 Prague, Czech Republic

## Abstract

**Background:**

Takotsubo cardiomyopathy (TC) aetiology has not been completely understood yet. One proposed pathogenic mechanism was coronary microvascular dysfunction (MVD). This study compared coronary flow and myocardial perfusion in patients with TC, microvascular angina (MVA), and a control group (CG).

**Methods:**

Out of 42 consecutive patients presented to our centre with TC from 2013 to 2017; we retrospectively selected 27 patients. We compared them with a sex- and age-matched group of 27 MVA cases and 27 patients with normal coronary arteries (CG). The flow was evaluated in the three coronary arteries as TIMI flow and TIMI frame count (TFC). Myocardial perfusion was studied with Blush-Score and Quantitative Blush Evaluator (QuBE).

**Results:**

TFC, in TC, revealed flow impairment in the three arteries compared to the CG (left anterior descending artery (LAD): 22 ± 8, 15 ± 4; *p* = 0.001) (right coronary artery: 12 ± 4, 10 ± 3; *p* = 0,025) (left circumflex: 14 ± 4, CG 11 ± 3; *p* = 0,006). QuBE showed myocardial perfusion impairment in the LAD territory in TC comparing with both the CG (8,9 (7,2–11,5) versus 11,4 (10–15,7); *p* = 0,008) and the MVA group (8,9 (7,2–11,5) versus 13,5 (10–16); *p* = 0,006).

**Conclusions:**

Our study confirmed that coronary flow is impaired in TC, reflecting a MVD. Myocardial perfusion defect was detected only in the LAD area.

## 1. Introduction

Takotsubo cardiomyopathy (TC) was described for the first time in the 1990 in Japan. This cardiomyopathy affects usually postmenopausal woman and it is often related to physical or emotional stress [1, 2].

TC presentation is “acute coronary syndrome-like” with chest pain, electrocardiographic alterations, left ventricle (LV) systolic dysfunction, and minimal troponin elevation and represents 1-2% of the ST-segment elevation myocardial infarction. Coronary angiography reveals normal or near-normal coronary arteries and the ventricle function usually recovers in a few weeks. The typical pattern of TC shows apical dysfunction (apical ballooning) at echocardiography and ventriculography, but other morphologies of TC are reported [3]. After more than 25 years after the first description, the aetiology and the pathogenesis of the TC are not completely understood yet. One of the possible mechanisms proposed is a microvascular dysfunction (MVD) with alteration in myocardial perfusion [4]. However different studies reported contrasting results about perfusion [5, 6].

Our study evaluated the role of MVD in TC assessing the flow and the perfusion at coronary angiography. We compared these results to another setting of supposed MVD like microvascular angina (MVA) and a control group (CG).

## 2. Methods

### 2.1. Population Characteristics

We retrospectively screened 42 consecutive patients with TC presented at our centre from October 2013 to January 2017. Out of 42 patients, we selected 27 cases. 15 patients were excluded because coronary angiography was not long enough to study myocardial perfusion with the blush score. Diagnosis of TC was achieved according to the presence of all 4 Mayo Clinic criteria [7]:

1. Transient hypokinesis, akinesis, or dyskinesis of the left ventricular mid segments with or without apical involvement: the regional wall motion abnormalities extend beyond a single epicardial vascular distribution
2. Absence of obstructive coronary disease or angiographic evidence of acute plaque rupture
3. New electrocardiographic abnormalities (ST-segment elevation and/or T-wave inversion) or modest elevation in cardiac troponin
4. Absence of pheochromocytoma or myocarditis.

27 age- and sex-matched patients with MVA were selected as a second group. MVA diagnosis was defined according to these features: typical chest pain according to the ESC guidelines [8], evidence of myocardial ischemia detected by perfusion test (myocardial scintigraphy), and normal coronary angiography.

The CG included 27 age- and sex-matched patients with atypical chest pain and normal coronary arteries at the angiography.

### 2.2. Angiographic Analysis

Angiograms were acquired with Philipp Allura FD 10 X-ray machine at 15 frames/sec using a manual injection of 5–10 mL of contrast. Iomeron 350 (Bracco Imaging Deutschland) or Ultravist 370 (Bayer Pharma) was used as a dye. For every patient the angiogram with no or minimal overlapping of the territories perfused by each vessel was selected and analysed offline. Qualitative assessment of epicardial coronary flow was measured using Thrombolysis In Myocardial Infarction (TIMI) flow criteria [9]. Quantitative evaluation of coronary flow was measured according to Gibson et al. using TIMI frame count [10] (TFC). TFC is the number of frames required, for contrast, to reach defined distal landmarks. The first frame to start counting was identified as the first frame in which dye fully fills the artery; the last frame considered is the one in which dye enters the end-point branch off the target artery [10].

### 2.3. Assessment of Myocardial Perfusion

Myocardial blush is the myocardial opacification resulting from injection of contrast into the coronary vessels and it depends on the microcirculation resistance if there is no evidence of obstructive (more than 70%) epicardial coronary stenosis. Qualitative assessment (blush score) was measured according to Van't Hof et al. [11]. Quantitative evaluation was achieved by Quantitative Blush Evaluator (QuBE) software [12]. This software indicates the status of myocardial perfusion with a score expressed in arbitrary units (“QuBE score”). The value of this index is calculated on an analysis performed on a manually selected polygonal area that surrounds the myocardial perfusion area of a vessel of interest. This area is automatically divided into blocks of 5 × 5 pixels, and for each block the 50% of the darkest pixels are evaluated, in order to calculate the value of a single frame. This process is then repeated for a maximum of 150 frames and a perfusion curve for the whole area is generated. The QuBE score finally reflects the filling and the emptying phase of the vessel; hence it corresponds to the sum of the maximum increase and the maximum decrease of the curve.

TIMI, TFC, blush grade, and QuBE were angiographically analysed by one interventional cardiology, a second assessment of all variables was repeated by a second observer blinded to the clinical data.

### 2.4. Statistical Analysis

Frequencies and percentages are given for quantitative variables. Variables were expressed as mean ± standard deviation (SD) or as median and interquartile range (IQR). Normality was tested by Shapiro-Wilk test. Categorical variables were expressed as percentages and analysed by two-tailed Fisher's exact test. The proportion of the categorical variables was compared using a chi-square analysis. Continuous variables were expressed as mean ± SD and compared with the Student's *t*-test or with the nonparametric Wilcoxon test. The interobserver agreement was evaluated with *t*-test or Wilcoxon. A *p* value of 0,05 was considered to be significant. Statistical analysis was performed by using Statistical Package for the Social Sciences version 22.0 (SPSS Inc., Chicago, Illinois).

This is an observational study; all patients provided informed consent for the coronary angiography due to clinical indication.

## 3. Results

### 3.1. Population Characteristics

Clinical characteristics of study population are presented in Table 1. Ejection fraction (EF) was lower in TC than in the others groups. However, at the time of discharge or at one-month follow-up echocardiography, a fully recovered EF was observed. In most of the patients with TC (85%) an apical pattern was observed. The remaining four patients (15%) showed a midventricular pattern at the time of the presentation.

Seventeen (63%) patients had severe reduction of ejection fraction and six of them required inotropic support for low cardiac output with a rate of in-hospital death of 11%.

### 3.2. Coronary Flow Assessment

**Figure 1:**
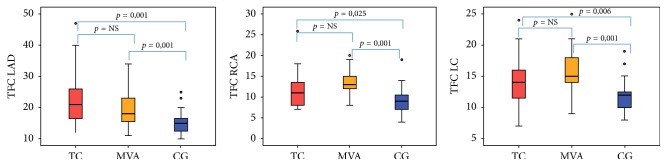
TIMI frame count in the LAD, RCA, and LC for the three groups. TC: Takotsubo cardiomyopathy; MVA: microvascular angina; CG: control group; TFC: TIMI frame count; LAD: left anterior descending coronary artery; RCA: right coronary artery; LC: left circumflex coronary artery; NS: nonsignificant.

Qualitative study of the TIMI flow in the LAD was lower in TC than in the CG (*p* = 0,01): indeed, TIMI 3 was observed in 70% of patients with TC versus 96% of the CG, while it was similar between MVA and CG. No differences were reported in TIMI flow on the right coronary artery (RCA) and left circumflex (LC) among groups (Table 2). Quantitative evaluation of TFC, in TC, was higher compared to the CG revealing flow impairment in all the three coronary vessels (LAD: TC 22 ± 8, CG 15 ± 4; *p* = 0.001) (RCA: TC 12 ± 4, CG 10 ± 3; *p* = 0,025) (LC: TC 14 ± 4 vs CG 11 ± 3; *p* = 0,006). TFC in MVA was comparable to TC and so higher compared to the CG in all the coronary arteries (LAD: MVA 20 ± 7, CG 15 ± 4; *p* = 0.001) (RCA: MVA 13 ± 3, CG 10 ± 3; *p* = 0,001) (LC: MVA 16 ± 4, CG 11 ± 3; *p* = 0,001) (Table 2, Figure 1). According to TFC LAD flow was reduced (mean value of 22) even among patients without apical pattern of TS (midventricular pattern).

No significant difference was reported between observers (*p* = 0,7).

### 3.3. Myocardial Perfusion Assessment

The qualitative evaluation of the blush score was significantly lower in the TC than in the other groups; a blush score of 3 was observed only in the 70% of the patients with TS versus all of them in the others groups (*p* = 0,001). No blush score differences were reported in the other coronary arteries among the three groups (Table 3).

**Figure 2:**
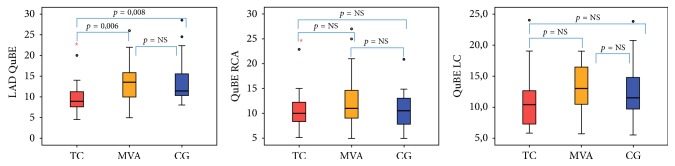
Quantitative blush assessment in the LAD, RCA, and LC for the three groups. TC: Takotsubo cardiomyopathy; MVA: microvascular angina; CG: control group; QuBE: Quantitative Blush Evaluator; LAD: left anterior descending coronary artery; RCA: right coronary artery; LC: left circumflex coronary artery; NS: nonsignificant. Red circle: mild outliers; red star: extreme outliers.

Similarly, the quantitative blush evaluation with QuBE software in TC group showed a significant impairment of myocardial perfusion in the LAD compared to the others groups (TC 8,9 (7,2–11,5) versus CG 11,4 (10–15,7); *p* = 0,008) (TC 8,9 (7,2–11,5) versus MVA 13,5 (10–16); *p* = 0,006). Analysing only the four patients without apical pattern of TS (midventricular pattern) reduced QuBE on the LAD territory (mean value of 9) was observed as well. QuBE analysis of the RCA and LC revealed analogue values in the three groups (Table 3, Figure 2).

No significant difference was reported between observers (*p* = 0,6).

## 4. Discussion

Pathophysiology of TC has not been completely clarified. Several hypotheses have been proposed: transitory epicardial coronary spasm, myocarditis, alterations of the coronary microcirculation, catecholamine excess, sympathetic nervous system hyperactivity in a setting of estrogenic deficiency, and genetic predisposition [3, 4, 13].

In our study, we retrospectively studied angiographic analysis of coronary flow and myocardial perfusion in patients with TC compared to MVA and a CG to assess the possible role of MVD in TC. We compared the flow and the perfusion using both qualitative (TIMI flow, blush score) and quantitative technique (TFC, QuBE). Our patients data were typical for TC [3] with a significant postmenopausal female prevalence (96%), recent physical or emotional stress (62%), and apical pattern in most of the patients (85%). Even if in a few weeks the recovery of LV function is usually achieved, the acute mortality rate of TC is around 4% [14] and in our population was even more with 3 (11%) in-hospital deaths. Moreover, six cases (22%) required inotropic support for acute heart failure. The TFC showed a coronary flow impairment in all the three vessel both in TC and in MVA compared to the CG. However, the qualitative eye-related TIMI flow evaluation was reduced only in the LAD, probably due to the inaccuracy of this method. Indeed, TFC is a more objective, reproducible, and sensitive technique to study the flow. Furthermore, TFC in TC was probably higher not only for MVD, but also for the acute hemodynamic conditions at the time of the angiography with low cardiac output requiring inotropic support in 6 cases, increased tele-diastolic pressure due to LV acute dysfunction, and compensatory increase of the heart rate.

We reported increased LAD TFC (mean value of 22) and reduced QuBE value on the LAD territory (mean value of 9) even in the four patients without apical pattern of TS (midventricular pattern). These findings suggested that the flow and the perfusion on the LAD territory are impaired regardless of the morphological pattern. Other papers described the mismatch between the akinetic territory and the area affected by the microcirculatory dysfunction [15]. However this observation could be proved by a study which compared the flow and the perfusion in different TS patterns with greater number of patients.

The coronary flow, as expected, was impaired in MVA patients for the underlying microvascular dysfunction.

Qualitative and quantitative data suggested that perfusion is impaired in TC compared with both the MVA and the CG, but only in the LAD region. No differences were reported in the blush and QuBE in the RCA and LC area in the three groups. Furthermore, the perfusion evaluated by QuBE in MVA patients was similar to the CG. This last data is probably due to the fact that the patients, at the time of the angiography, were at rest, in optimal medical therapy, and that the jeopardized area of ischemia, typical of MVA, did not allow finding out alteration in the blush with QuBE even if it is probably present.

According to the literature, controversial results about TFC in TC are reported: Khalid et al. observed that, in 16 TC patients, TFC was higher compared to controls in LAD artery, but not in the other vessels [16]; another paper, as we did, showed that TFC increased in all the three vessels of 16 cases with TC compared to healthy subjects [17]. Fazio et al. found out mixed results on 24 cases of TC with 9 patients with a flow impairment in all the coronaries, 6 in two vessels, and 9 only in one [15]. De Caterina et al. selected 25 TC patients with apical pattern and reported flow and perfusion impairment in the LAD compared to a control group and ST-segment elevation patients after successful reperfusion [18]. Microvascular dysfunction in TC and its reversibility were also confirmed with other techniques like positron emission tomography [19] and contrast transthoracic Doppler echocardiography [20]. However, another study evaluated the coronary vascular reactivity with pharmacological test in 10 TC patients, suggesting that MVD and consequent impaired vascular function are a chronic condition in TC and not a transient phenomenon [20].

Our data confirmed deterioration of the epicardial flow in TC that it is probably due to MVD with a significant impairment of myocardial perfusion in LAD territory and this finding could not be related to morphological pattern of TC presentation.

Our study presents some limitations: (1) the small number of patients was selected, but TC is a rare disease, representing only 1-2% of ST-segment elevation myocardial infarction, and we excluded some patients because the angiography was not long enough for the blush score evaluation; (2) we cannot be conclusive on the reversibility of MVD because we did not perform a control coronary angiography after the acute phase, but all our cases showed a recovery of LV function; (3) the study is retrospective and we cannot be conclusive, but our data are well supported by several other evidences; (4) we did not perform cardiac magnetic resonance, the gold standard to assess MVD. However, as proved by Porto et al. [21], microvascular impairment detected by QuBE software is accurate and strongly correlated to magnetic resonance data.

In conclusion, our study showed that the epicardial coronary flow is impaired in TC and in MVA compared to a CG, reflecting a MVD. However, it seemed to be related to myocardial perfusion defect only in the LAD area as proved by QuBE.

## Figures and Tables

**Table 1:** Baseline characteristics of the patients.

	TC (*N* = 27); *n* (%)/mean ± SD	MVA (*n* = 27); *n* (%)/mean ± SD	CG (*n* = 27); *n* (%)/mean ± SD	*p* value (TCvsCG/TCvsMVA/MVAvsCG)
Age	67 ± 10	65 ± 9	64 ± 9	NS^*∗*^
Female	23 (85%)	23 (85%)	23 (85%)	NS^*∗*^
Caucasian	27 (100%)	27 (100%)	27 (100%)	NS^*∗*^
Hypertension	15 (55%)	17 (62%)	20 (74%)	NS^*∗*^
Diabetes Mellitus	3 (11%)	5 (18%)	3 (11%)	NS^*∗*^
Hypercholesterolemia	15 (55%)	13 (48%)	16 (59%)	NS^*∗*^
Current Smoking	10 (37%)	6 (22%)	8 (30%)	NS^*∗*^
EF, %	35 ± 9	58 ± 7	57 ± 10	0,001/0,001/NS
Menopause	26 (96%)	/	/	/
Emotional trigger	6 (22%)	/	/	/
Physical trigger	11 (40%)	/	/	/
Apical pattern	23 (85%)	/	/	/
LVOT obstruction	3 (11%)	/	/	/
Troponin	355 ± 362	/	/	/
ST-segment elevation	11 (40%)	/	/	/
Prolonged QTc^∨^	9 (33%)	/	/	/
EF ≤ 35%	17 (63%)	/	/	/
Inotropic support	6 (22%)	/	/	/
In-hospital death	3 (11%)	/	/	/

TC: Takotsubo cardiomyopathy; MVA: microvascular angina; CG: control group; LVOT: left ventricle outflow tract obstruction; EF: ejection fraction. NS: nonsignificant; ^∨^QT corrected interval above 450 ms in males or above 470 ms in females; *∗* indicates the same result for all the three matches.

**Table 2:** Coronary flow evaluation in the three groups.

	TC (*N* = 27); *n* (%)/mean ± SD	MVA (*n* = 27); *n* (%)/mean ± SD	CG (*n* = 27); *n* (%)/mean ± SD	*p* value (TCvsCG/TCvsMVA/MVAvsCG)
TIMI 3 LAD	19 (70%)	22 (81%)	26 (96%)	**0,01**/NS/NS
TFC LAD	22 ± 8	20 ± 7	15 ± 4	**0,001**/NS/**0,001**
TIMI 3 RCA	27 (100%)	27 (100%)	27 (100%)	NS^*∗*^
TFC RCA	12 ± 4	13 ± 3	10 ± 3	**0,025**/NS/**0,001**
TIMI 3 LC	27 (100%)	25 (93%)	27 (100%)	NS^*∗*^
TFC LC	14 ± 4	16 ± 4	11 ± 3	**0,006**/NS/**0,001**

TC: Takotsubo cardiomyopathy; MVA: microvascular angina; CG: control group; LAD: left anterior descending coronary artery; RCA: right coronary artery; LC: left circumflex coronary artery; *∗* indicates the same result for all the three matches.

**Table 3:** Myocardial perfusion evaluation in the three groups.

	TC (*N* = 27); *n* (%)/median (interquartile range)	MVA (*n* = 27); *n* (%)/median (interquartile range)	CG (*n* = 27); *n* (%)/median (interquartile range)	*p* value (TCvsCG/TCvsMVA/MVAvsCG)
Blush LAD	19 (70%)	27 (100%)	27 (100%)	**0,001**/**0,001**/NS
QuBE LAD	8,9 (7,2–11,5)	13,5 (10–16)	11,4 (10–15,7)	**0,008**/**0,006**/NS
Blush 3 RCA	26 (96%)	27 (100%)	27 (100%)	NS^*∗*^
QuBE RCA	10 (7,7–12,4)	11 (9–15,4)	10,5 (7,7–13)	NS^*∗*^
Blush 3 LC	27 (100%)	26 (96%)	27 (100%)	NS^*∗*^
QuBE LC	10,4 (7,1–12,8)	13 (10–17)	11,5 (9,5–15)	NS^*∗*^

TC: Takotsubo cardiomyopathy; MVA: microvascular angina; CG: control group; TFC: TIMI frame count; QuBE: Quantitative Blush Evaluator; LAD: left anterior descending coronary artery; RCA: right coronary artery; LC: left circumflex coronary artery; *∗* indicates the same result for all the three matches.

## Abbreviations

TC: Takotsubo cardiomyopathy
MVD: Microvascular dysfunction
MVA: Microvascular angina
CG: Control group
TFC: TIMI frame count
QuBE: Quantitative Blush Evaluator
LAD: Left anterior descending artery
RCA: Right coronary artery
LC: Left circumflex coronary artery
EF: Ejection fraction.
